# Supplementary material for: Orbital actinomadura madurae actinomycetoma: Case report and literature review
Source: PLoS Negl Trop Dis. 2025 Apr 29;19(4):e0013035. doi: 10.1371/journal.pntd.0013035 (PMC12040158; doi:10.1371/journal.pntd.0013035)
Supplement: Attachment — Submitted filename: Reviwers comments 2025.pdf [file pntd.0013035.s003.pdf]

Dear Editor

Ref. Orbital Actinomadura madurae Actinomycetoma

Comments:

1. Revise the title. For example. Orbital Actinomadura madurae Actinomycetoma: case report and literature review

Author reply: Taken in consideration and re titled.

2. Review the instructions to PLOSNTD authors, Research Articles should include an 'Author summary' section. Brief description of the case.

Author reply: Was done

3. Include the CARE Checklist as supplementary material. In addition, you should ensure that you comply with each of its items.

Author reply: We comply with the items

4. Include an Introduction section with a brief paragraph from the literature review, including the objective of the case.

Author reply: Was done

5. Include an Ethical Statement section. For example. This study was approved by the ethics committee of XXXXXXX and the study participant was informed about the study procedures and written informed consent was obtained.

Author reply: Was done

6. Submit the original images in high quality to improve the quality of the report.

7. Case Report section. Describe briefly whether a differential diagnosis was considered in the case.

Author reply: The images provided were the original, due to the war conflict in Sudan the resolution was weak.

8. Your manuscript is missing the following sections: Results, and Methods. Please ensure all required sections are present and in the correct order. Make sure section heading levels are clearly indicated in the manuscript text, and limit sub-sections to 3 heading levels

Author Reply: Our manuscript is a case presentation.

Journal Requirements:

Author reply: **All journals requirements were made.**

Ali Awadallah Saeed

Coressponding author
