# Supplementary material for: Orbital actinomadura madurae actinomycetoma: Case report and literature review
Source: PLoS Negl Trop Dis. 2025 Apr 29;19(4):e0013035. doi: 10.1371/journal.pntd.0013035 (PMC12040158; doi:10.1371/journal.pntd.0013035)
Supplement: Attachment — Submitted filename: Response to editor April 2025.docx [file pntd.0013035.s004.docx]

Dear Editor

Ref. Orbital Actinomadura madurae Actinomycetoma

Thank you for your feedback. As this is a case study, the structure differs slightly from traditional research articles.

**Ali Awadallah Saeed**

**Coressponding author**
